# The psychometric properties and the factorial structure of COVID-19 Vaccines Acceptance scale (VAC-COVID-19) within the Arabic language in a Palestinian contex

**DOI:** 10.1186/s12889-022-14229-y

**Published:** 2022-09-29

**Authors:** Fayez Mahamid, Guido Veronese

**Affiliations:** 1 Psychology and Counseling Department, An-Najah National University, Nablus, Palestine; 2 University of Milano, Bicocca, Italy

**Keywords:** Vaccination acceptance, COVID-19, Test validation, Palestine

## Abstract

**Background:**

The COVID-19 Vaccines Acceptance scale (VAC-COVID-19) is an international measure designed to evaluate vaccination acceptance against the COVID-19 virus. The current scale was translated from English to Arabic and validated within the Palestinian context.

**Aims:**

Our study aimed to test the factorial structure and the psychotic properties of the VAC-COVID-19 within the Palestinian context using exploratory factor analysis (EFA) and confirmatory factor analysis (CFA) through 484 participants selected using online method techniques.

**Findings:**

The VAC-COVID-19 was a reliable and valid method in assessing COVID-19 vaccine acceptance among Palestinians. Results of CFA indicated a stable construct of a two-factor solution in assessing COVID-19 vaccination acceptance in a Palestinian context. (1) Reasons for not receiving the vaccination, and (2) for receiving the vaccination.

**Conclusion:**

The VAC-COVID-19 was a valid method to assess vaccination acceptance in the Arabic language within the Palestinian context. Therefore, it is recommended to conduct similar studies with diverse samples in Palestinian society; it would be prudent to target at-risk populations needed to develop the scale and its factorial structure. The VAC-COVID-19 can be a useful measure to assess vaccination acceptance among Palestinians, enabling health providers to implement interventions to modify negative attitudes toward not receiving vaccinations.

## Theoretical Background

The coronavirus disease of 2019 (COVID-19) remains a current threat to public health [26]. Across the world, several countries implemented large-scale measures to reduce the rapid spread of COVID-19, such as strict social distancing guidelines and limitations on movement, otherwise known as ‘lockdowns’ [19]. However, despite these measures, the pandemic is still ongoing. While government mandates of personal protective gear, such as masks, are vital to managing the spread of this infectious disease, vaccination may provide more extraordinary safety measures against hospitalizations related to the COVID-19 virus [18].

By April 22, 2022, about 11.2 billion COVID-19 vaccines had been administered in more than 197 countries. The percentage of individuals fully vaccinated worldwide is 60% of the total world population. The rate of receiving the COVID-19 vaccine varied in different countries; for example, in China, 58%, USA 66%, New Zealand 83%, Saudi Arabic 72%, Jordan 44%, Egypt 33%, and Lebanon 32%. In Palestine, the percentage of fully vaccinated people is 38% [26].

The WHO Strategic Advisory Group of Experts (SAGE) defines vaccine hesitancy as a delay in acceptance or refusal of vaccination despite the availability of vaccination services [5]. Complacency, convenience, and confidence are considered factors that affect one’s attitude towards vaccination acceptance [27]. Complacency signifies a low perception associated with the risk of getting the disease and, therefore, deems the need for vaccination unnecessary. Convenience looks at vaccines’ affordability, availability, and delivery in a reliable context. Confidence refers to the trust in vaccination safety and effectiveness, as it relates to the competence of the healthcare systems [25].

Earlier studies have shown vaccine hesitancy to be a global phenomenon, with variability in the cited reasons owing to the refusal of vaccine acceptance. The prevailing reasons include anticipated benefits vs. risks, a lack of knowledge and/or awareness, and particular religious beliefs [14, 27, 26). A global survey to determine possible rates and factors related to accepting a COVID-19 vaccine was administered to 13,426 people in 19 countries. Of those surveyed, 71.5% stated that they would be ‘very’ or ‘somewhat likely to take a COVID-19 vaccine. Participants who responded with high levels of trust in government sources and information were ‘more likely to listen to their employer’s advice and take the vaccine [17].

One study had participants from Jordan complete a survey to investigate the acceptability, predictors, and perceived beliefs toward the COVID-19 vaccines. A total of 3,100 participants were involved in the study, and vaccination acceptance among participants was reasonably low (37.4%); the findings also showed that those who take the seasonal influenza vaccine (2.03%) were more inclined to take the COVID-19 vaccines, as well as those who were willing to pay for vaccines (19.22%), and who believed that vaccines are generally safe (9.25%), [11].

Few studies have centred on the demand for vaccines in middle and lower-income countries (LMICs). This may be due to varying factors involving the population compared to higher-income countries. LMICs may have fewer means when introducing new vaccines and may need to contend with citizenry who show hesitancy due to their beliefs [13].

Studies from different cultures have identified many factors influencing the acceptance of the COVID-19 vaccine. [8] investigated the knowledge, attitudes, and vaccine acceptance/hesitancy towards COVID- vaccinations in Italy. Factors significantly associated with willingness to receive the COVID-19 vaccination were confidence in vaccines, fear of contracting COVID-19 infection, considering vaccination to be the best strategy to counteract the COVID-19 virus, and adherence to influenza vaccination during the 2020/2021 season[21] evaluated psychological characteristics associated with COVID-19 vaccine hesitancy and resistance in Ireland and the United Kingdom. Results showed that vaccine-hesitant/resistant respondents in Ireland and the U.K. were similar across various psychological constructs. In both populations, those resistant to a COVID-19 vaccine were less likely to obtain information about the pandemic from traditional and authoritative sources and had similar levels of mistrust in these sources compared to vaccine-accepting respondents. [25] study aimed to evaluate COVID-19 vaccine acceptance in Jordan. This study showed the high prevalence of COVID-19 vaccine hesitancy and its association with conspiracy beliefs among university students in Jordan. Moreover, dependence on social media platforms was significantly associated with lower intention to get COVID-19 vaccines compared to dependence on medical doctors, scientists, and scientific journals.

Therefore, information concerning individuals’ attitudes and considerations for vaccinating should be investigated to encourage individuals to take the vaccine and allow communities to uptake their vaccination rates [10].

The COVID-19 vaccine acceptance scale (VAC-COVID-19) is a new international instrument developed by [22] to explore individuals’ attitudes and considerations in vaccinating against COVID-19 in Peru. According to CFA and EFA results, two subscales with 11 items were found to explain 58.17% of the total variance; the two subscales can be used separately in assessing reasons for not receiving the vaccination and reasons for receiving a vaccination, or they can be used together after reversing the scoring for one of these subscales. Each item had five possible Likert-type responses (strongly disagree = 1 score, disagree = 2 scores, neither disagree nor agree = 3 scores, agree = 4 scores, and strongly agree = 5 scores). The fit indices show that the proposed model is adequate. Finally, Cronbach’s α was very satisfactory for the generated scale.

Several studies have been implemented to validate vaccination hesitancy scales in different contexts. [24] tested the psychometric properties of a modified version of the Vaccine Hesitancy Scale (VHS) among people with HIV in the United States. Results illustrate that the modified VHS for COVID-19 vaccination has adequate psychometric properties to assess vaccination hesitancy. [28] examined whether DrVac-COVID19S is measurement invariant across different subgroups (Taiwanese vs. mainland Chinese university students; males vs. females; and health-related program majors vs. non-health-related program majors. The findings indicated that the DrVac-COVID19S is a stable method across the subgroups. [1] assessed the psychometric properties of the 5 C scale for assessing the COVID-19 vaccine’s psychological antecedents in the Arabic language. Results revealed that the Arabic version of the 5 C scale is a valid and reliable tool to assess the psychological antecedents of the COVID-19 vaccine among the Arab population. [7] examined the validation indicators of an Italian version of the Vaccination Attitudes Examination (VAX) scale; the results showed that the VAX-I scale appears to be a valid instrument to assess vaccine hesitancy in the Italian context. Finally, [16] tested the validation of the Multidimensional Covid-19 Vaccine Hesitancy Scale (CoVaH) in the Hungarian context, the CoVaH displayed excellent fit indices and internal consistencies and was found to have good validity in identifying Covid-19 vaccine hesitancy in the general population.

Despite the validation of different vaccination hesitancy scales in various contexts, there is a need to explore the VAC-COVID-19 scale’s validity and reliability indicators in a specific cultural context, such as within the Palestinian context [19, 20].

Hence, this is the first study to explore the validity and reliability indicators of VAC-COVID-19 within the Palestinian population. Therefore, our study would test the VAC-COVID-19’s two-factor structure in assessing vaccination acceptance among Palestinians, (b) the VAC-COVID-19 would be a reliable measure in assessing reasons for not receiving and receiving vaccination in Palestine, (c) the VAC-COVID-19 would be a valid measure in assessing reasons for not receiving and receiving vaccination in Palestine.

## Methods

### Participants

Participants were recruited using emails, Instagram, Twitter, and WhatsApp. The sample size for this study was calculated based on 95% CI and a 5% margin of error using the Raosoft software sample size calculator; the recommended sample was 484 participants. Regarding the academic qualification of participants, 36.8% were M.A. holders, and 63.2% were B.A. holders. Sample distribution by gender shows that 31.6% of participants were males and 68.4% were females. 73.8% of participants were from cities, and 26.2% were from villages. Finally, 33.9% of participants were aged 22–33, 25.6% were aged 34–43, 25.6% were aged 44–51, and 15.1% of participants aged 51 years or more. Inclusion criteria in our study required participants to be: (1) Native Arabic speakers, (2) Palestinian, and (3) free from psychotic and neurodevelopmental disorders.

### Instruments

#### The COVID-19 Vaccines Acceptance scale (VAC-COVID-19)

The VAC-COVID-19 Scale is a self-report developed by [22] to assess participants’ beliefs and behaviours, and attitudes toward vaccination. The scale ended up with 11 items with two main sub-factors: Factor 1 (reasons for not receiving vaccination), the items (1–7) represent this factor, while Factor 2 (reasons for receiving vaccination), represented by items (8–11). The responses to the items are interpreted on a 4-point Likert scale ranging from 1 to 4 (1 = never, 2 = sometimes, 3 = often, 4 = always).

#### The fear of Coronavirus-19 scale (FCV-19 S)

The FCV-19 S is seven items self-report scale designed to test fear related to the COVID-19 pandemic. The measure assesses emotional and behavioural responses to the Covid-19 pandemic. In order to repose to the scale items, participants are requested to range their responses on a five-item Likert scale ranging from 5 ( strongly agree) to 1( strongly disagree). The total score on the scale ranged from 7 to 35, with a higher degree indicating a high level of fear related to the COVID-19 pandemic [2],[20] validated the scale with the Palestinian context. The scale showed excellent indicators of validity and reliability.

#### The Drivers of COVID-19 Vaccination Acceptance Scale (DrVac-COVID19S)

The DrVAC-COVID19S scale is 12 items self-report scale designed to assess beliefs on the effects of vaccinations uptake, care about vaccination uptake, the role of COVID-19 vaccination in preventing infection, and the level of confidence in getting COVID-19 vaccination. In order to respond to the scale items, participants are requested to range their responses on a five-item Likert scale ranging from 5 ( strongly agree) to 1( strongly disagree), with a high score on the.

DrVac-COVID19S indicates a higher degree of COVID-19 vaccine acceptance [28].

### Research procedures

The current study was conducted in January of 2022 and targeted Palestinians in the West Bank and Gaza. The sample of our study was recruited using online methods techniques. Participants were given information to make an informed decision regarding the study, followed by a signed informed consent. Additionally, participants were informed of the purpose of the research and a brief description of the study instruments. The VAC-COVID-19 was translated from English into Arabic; then, a translated version was evaluated by five Arab professionals who are experts in Counseling, Psychology, Education, and the Arabic Language. The professionals assessed the relevance and clarity of the translated questions. Following completion, the translated questionnaire draft was back-translated into English by an independent professional. The translated version was distributed amongst 80 participants as a pilot test; comments were then utilized to further refine the questionnaire for clarity. We also calculated test-retest reliability for the VAC-COVID-19 scale. The measure was re-administered to participants (Reliability sample) after three weeks of the first administration, and the correlation between the VAC-COVID-19 scores at times one and two was assessed.

### Data Analysis

We used Pearson’s Correlation Coefficient to test the correlation between VAC-COVID-19, DrVac-COVID19S, and FCV-19 S. Moreover, the Independent samples t-test was used to test the differences due to study demographic variables separately (Gender, Academic level, and residency). Cronbach’s Alpha, Guttmann Split-Half, and test-retest were calculated to explore the reliability indicators of the scale using Statistical Package for Social Sciences, SPSS 28.

In order to test the CFA model, AMOS 25 software was used. The goodness of fit index (GFI), the Normed Fit Index ( NFI), the Incremental fit index ( IFI), and the root mean square error of approximation (RMSEA) were tested following [14] criteria, who mentioned that RMSEA values should be less than 0.07, and the NIF, GfI, and IFI values should be more than 0.90.

## Findings

### Exploratory factor analysis

Exploratory factor analysis (EFA) indicated a 2-factor solution of the VAC-COVID-19 in a Palestinian context; the two factors explained 72.08 of cumulative variance. The eigenvalues of the two factors were as follows: 42.24 and 29.84.

### Confirmatory factor analysis

**Fig. 1:**
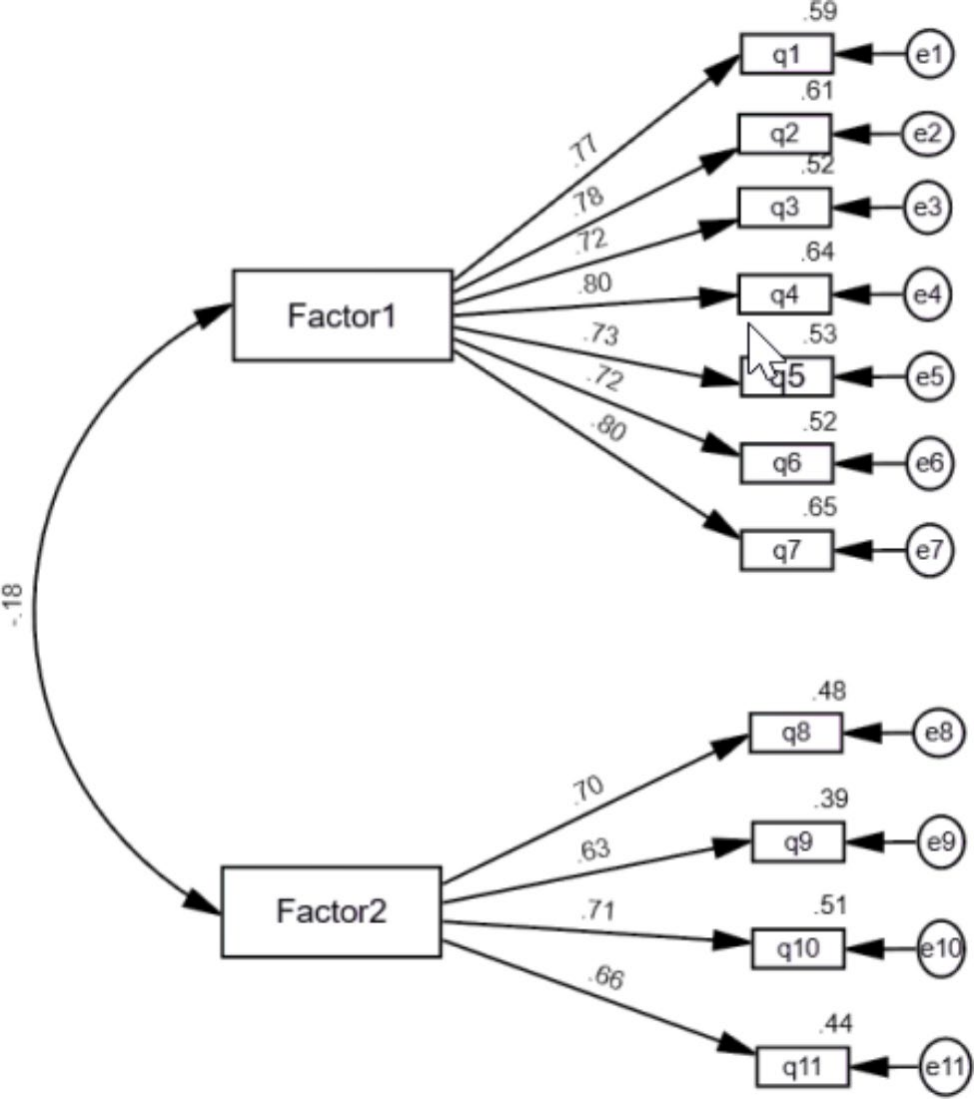
Confirmatory factor analysis of Vaccines Acceptance Scale (VAC COVID-19) within Palestinian context

Our findings revealed that 11 items fit together conceptually and positively correlated with the total score of VAC-COVID-19. In Table 1, Amos’ model yielded two main factors: (1) reasons for not receiving a vaccination and (2) for receiving the vaccination. The results (see Fig. 1) demonstrated good indices values with a good model of fit (CFI = 0.95, GFI = 0.95, NFI = 0.89, RFI = 0.88, IFI = 0.89, and RMSEA = 0.07).

**Table 1:** Covariance created in the two-scale construct (n **=** 484)

Estimate	Standardized	S.E.	C.R.	***P***
Reasons for not receiving vaccination	2.29	0.03	71.74	***0.001
Reasons for receiving the vaccination	3.19	0.02	115.49	***0.001

***P significance ≤ 0.001

### Concurrent validity

A Person Correlation Coefficient was calculated between the VAC-COVID-19, DrVac-COVID19S, and FCV-19 S to determine if the scale can evaluate vaccine acceptance in the Palestinian context, as presented in Table 2.

**Table 2:** Pearson Correlation between VAC-COVID-19, DrVac-COVID19S, and FCV-19 S (n = 484)

Scale	**1**	**2**	**3**
1. VAC-COVID-19	1	. 62**	0.23**
2. DrVac-COVID19S		1	0.26**
1. FCV-19 S			1

**correlation is significant at the 0.01 level (2-tailed)

The VAC-COVID-19 correlated significantly with DrVac-COVID19S (r = .62; *p* < .01), moreover, a positive correlation was found between VAC-COVID-19 and FCV-19 S (r = .23; *p* < .01). Finally, DrVac-COVID19S correlated positively with FCV-19 S (r = .26; *p < .01*).

### Reliability of PSAS Scale

To test the reliability of the VAC-COVID-19 scale, test-retest, Guttmann Split-Half and Cronbach’s alpha measures were calculated as presented in Table 3.

**Table 3:** Reliability analysis of VAC-COVID-19 (n = 484)

No.	Items	Cronbach’s Alpha if Item Deleted	Corrected Item-Total Correlation	Test-retest	Guttmann Split- Half	α
1	I think they are going to insert electronic chips/transistors to control my brain	0.86	0.68	0.83		
2	I think SARS-CoV-2 vaccines are part of the plan of a large company that created COVID-19	0.86	0.70	0.81		
3	I think some SARS-CoV-2 vaccines can come from a former communist republic (like Russia), which may influence communist thinking. may result in influences on communist thinking	0.87	0.62	0.82		
4	I have worried about the bond I have with my baby	0.86	0.72	0.84		
5	I think COVID-19 is an invention of the World Health Organization (WHO) or other similar institutions	0.88	0.62	0.82		
6	A healthy life is enough to fight disease.	0.89	0.79	0.83		
7	I do not trust my health care system (including healthcare personnel)	0.86	0.71	0.84		
8	I want to get back to the life I had before the pandemic	0.84	0.68	0.83		
9	15 SARS-CoV-2 vaccines should contribute to improving the health of my family or loved ones	0.85	0.70	0.80		
10	I think SARS-CoV-2 vaccines should contribute to improving the health of the community/population.	0.81	0.77	0.86		
11	I do not want to wear personal protective equipment anymore (masks).	0.85	0.72	0.81		
	Reasons for not receiving the vaccination			0.81	0.83	0.84
	Reasons for receiving the vaccination			0.80	0.81	0.83
	Total score of VAC-COVID-19			0.82	0.84	0.88

Cronbach’s Alpha Coefficient of VAC-COVID-19 indicated a high level of internal consistency (α = 0.88). Moreover, the split-half coefficient also showed a high degree of reliability (0.84). In order to calculate test-retest reliability for VAC-COVID-19, the scale was re-administered to 80 participants after three weeks of the first administration. The correlation between the VAC-COVID-19 scores at times one and two was 0.82, indicating that VAC-COVID-19 is a stable measure to test vaccination acceptance.

Results of Table 4 show no significant differences between MA holders and BA holders on factor one, not receiving vaccination (t = 1.12; *p > .05*) and factor two, receiving vaccination (t = 0.47; *p > .05*). Moreover, significant differences were found between males and females on not receiving vaccination dimension in favor of females (t = 2.60; *p ≤ .01*), while no significant difference were noted between males and females on receiving vaccination dimension (t = 0.52 *p > .05*). Finally, no significant differences were found between city residents and village residents on not receiving vaccination (t = 0.67; *p > .05*), and receiving vaccination (t = 1.56; *p > .05*).

**Table 4:** Differences in vaccine acceptance by academic level, gender, and residence (n = 484)

dependent variable	Variable	Academic level		Gender		Residence	
		**MA**	**BA**	**Male**	**Female**	**City**	**Village**
Factor 1	Mean (SD)	2.33(0.70)	2.26(0.69)	2.17(0.67)	2.35(0.70)	2.28(0.70)	2.34(0.67)
Factor 2	Mean (SD)	3.20(0.60)	3.18(0.60)	3.17(0.61)	3.20(0.60)	3.21(0.59)	3.09(0.62)

## Discussion

The current study aimed to test the factorial structure and the psychometric properties of VAC-COVID-19 within the Arabic language in a Palestinian context. VAC-COVID-19 is an international instrument designed to test Covid-19 vaccination acceptance. The findings of our study indicated that the VAC-COVID-19 scale is a valid and reliable tool in exploring reasons for not receiving and receiving vaccination among Palestinians; a positive correlation was found between VAC-COVID-19 and other measures; DrVac-COVID19S and FCV-19 S which were designed to test fear of COVID-19 and vaccination acceptance. Results of EFA and CFA showed a stable construct of a two-factor solution in assessing vaccination acceptance among Palestinians. The original two factors of VAC-COVID-19 are (1): Reasons for not receiving a vaccination and (2) Reasons for receiving the vaccination. Recent studies tested vaccination acceptance, showing that these two constructs are theoretically relevant. For example, [6], who reviewed 35 studies that tested factors affecting Covid-19 vaccination hesitancy among health care workers, found that the top reasons for COVID-19 vaccination hesitancy were vaccine efficacy, safety and potential side effects.

[4] tested vaccination acceptance and associated vectors among Jordanians; the results indicated that the main reasons for vaccination refusal were the lack of trust and concerns regarding vaccine use. [23] evaluated the acceptability of COVID-19 vaccination among health providers in Greek; the results indicated a high level of vaccination acceptance against COVID-19 among health providers.

Our findings indicated that VAC-COVID-19 is an excellent measure of vaccination acceptance in the Palestinian context. Validating a new international instrument in a context such as Palestine is crucial to clinical practices and mental health services, given that Palestinian people suffer from high anxiety levels and different environmental stressors. The prolonged political conflict between Israelis and Palestinians creates a sense of overconfidence and insecurity among Palestinians, leading to refusing vaccinations that may be seen as a conspiracy targeting them.

The Palestinian Authority returned all vaccinations with a short expiration date to Israelis. The incident exemplifies the lack of trust between the two entities, even when fundamental values for both parties, such as human rights and public health, are at stake [9]. Such events and others, the Israeli occupation, and political violence in the Palestinian community have made the Palestinians refuse to take vaccinations and doubt their usefulness from a medical point of view.

Therefore, validating new instruments to explore Palestinian viewpoints toward vaccination will help mental health providers assess reasons for receiving and not receiving the vaccination, leading to interventions to modify negative attitudes toward vaccination. Irrational thoughts and false beliefs about vaccinations can negatively affect health and mental health among Palestinians; accordingly, the biggest challenge that faces mental health providers is to identify these false and irrational beliefs about receiving vaccinations and modify them.

Our findings showed significant differences between males and females in not receiving vaccinations favouring females. This result may be explained in light of the spread of many rumours in Palestinian society about the possible adverse effects of vaccines on pregnancy and future childbearing. Several studies were designed to explore gender differences in willingness to receive the COVID-19 vaccine; for example, [15] found that the percentage of women in Japan who were willing to receive the COVID-19 vaccine was lower than among men (33.0% vs. 41.8%). [12] tested gender differences in accepting to receive the COVID-19 vaccine in Israel; the results indicated that among men, 51.3% agreed to receive the vaccine, compared with only 25.6% of women. [3] explored attitudes toward receiving the COVID-19 vaccination; the results found that women were more likely to refuse the vaccine. Reliable instruments such as *VAC-COVID-19* will contribute in shedding light on gender differences in vaccinal choices and attitudes.

### Limitations of the study

Testing a new measure’s psychometric properties and factorial structure is an ongoing process; the present study has several limitations that may offer opportunities for future research to continue testing the VAC-COVID-19 within different contexts. First, the study targeted an academic convenience sample (B.A. and M.A. holders) using self-report methods, focusing on different samples in Palestinian society to test the scale’s psychometric properties and required factorial structure. Second, our study data was collected during political events in the West Bank of Palestine, which may skew the scale’s psychometric properties and factorial structures; testing the psychometric properties of VAC-COVID-19 within different periods is needed. Third, our sample does not sufficiently represent different sub-groups and regions in Palestine. Testing the scale’s psychometric properties within more at-risk groups in Palestine is necessary. Finally, we used the DrVac-COVID19S scale to test the concurrent validity of VAC-COVID-19. The psychometric properties of this tool are not proved within the Arabic language in a Palestinian context, indicating the need to validate this scale in a Palestinian context to increase the robustness of the results.

## Conclusion

To our knowledge, the VAC-COVID-19 is the first instrument validated in the Palestinian context to test vaccination acceptance. The VAC-COVID-19 was a valid and reliable measure to assess reasons for not receiving and reconvening vaccination within the Palestinian context. The two-factor solution of the VAC-COVID-19: (1): Reasons for not receiving vaccination (2) Reasons for receiving vaccination fit the data reasonably well in a confirmatory-factor analysis. It is recommended to conduct similar studies with diverse samples in the Palestinian society; it would be prudent to target at-risk populations needed to develop the scale and its factorial structure. The VAC-COVID-19 can be a useful measure to assess vaccination acceptance among Palestinians, enabling health providers to use the scale in assessing individuals’ attitudes and perceptions toward vaccination acceptance.

## Data Availability

The datasets generated during and/or analyzed during the current study are available from the corresponding author on reasonable request.

## Abbreviations

VAC-COVID-19: COVID-19 Vaccines Acceptance scale.
EFA: Exploratory factor analysis.
CFA: Confirmatory factor analysis.
LMICs: lower income countries.
VCS: Vaccine Hesitancy Scale.
VAX: Vaccination Attitudes Examination.
FCV-19S: The Fear of Coronavirus-19 Scale.
DrVac-COVID19S: The Drivers of COVID-19 Vaccination Acceptance Scale.
GFI: The goodness of fit index.
NFI: The Normed Fit Index.
IFI: the Incremental fit index.
RMSEA: The root mean square error of approximation.
SD: Standard deviation.
α: Cronbach’s alpha for reliability.
